# Supplementary material for: A Novel Facet of In-Hospital Food Consumption Associated with Hospital Mortality in Patients with Scheduled Admission—Addition of a Study Protocol to Test the Existence of Effects of COVID-19 in the Same Study in the Post-COVID-19 Period
Source: Nutrients. 2024 Jul 19;16(14):2327. doi: 10.3390/nu16142327 (PMC11280368; doi:10.3390/nu16142327)
Supplement: Supplementary file 1 [file nutrients-16-02327-s001.zip › nutrients-3109991-supplementary.pdf]

Supplementary Table S1. The collected data and their timing of collection during hospitalization are shown.

Compared data	Data collected	Timing of data collection		
		At admission	during hospitalization	At discharge
Demographics	Sex, age, height, body weight, BMI, admitted word	●		
	CCI score , ADL, number of drugs	●		
	Type of admission, Scheduled or Emergency	●		
	Diagonosis by ICD-10 version 2019	●		
	LOS before Study Day		● (on nDay)	
Blood test	CRP: throughout the hospital stay		●	
Nutritional parameters	Oral intake, yes or no		●	
	% provided food intake (full, 75%, 50%, 25%, none)		●	
	Food texture by IDDSI, regular or non-regular		●	
Outcome measures				
Primary outcome	In-hospital mortality		●	
Secondary outcomes	Length of hospitalization, days		●	
	The highest CRP throughout the hospital stay		●	
	Days from admission to the day of the highest CRP		●	
	Survival within 30 days after hospitalization, yes or no			●
	Living place after discharge, home or other than home			●

Supplementary Table S2. The list of nutritional assessment tools used for COVID-19 patients and their reference number. The list is arranged in descending order of the number of papers that reported nutritional assessment tools used in COVID-19 patients during the COVID-19 pandemic, and their reference paper numbers are shown. Details of the reference papers are given in the supplementary table. From this table, figure 2 was graphically made.

Nutritional assessment tool	Reference
**NRS 2002**	**26 - 42**
**MUST**	**28, 30, 42 - 47**
**MNA-SF**	**28, 30, 48 - 51**
**GLIM**	**26, 50 - 53**
**PNI**	**54 - 56**
**GNRI**	**57 - 59**
**mNUTRIC**	**59 - 61**
**SNAQ**	**62, 63**
**SARC-F**	**43, 62**
**CONUT**	**57, 64**
**mNRS**	**30**
**R-MAPP**	**46**
**SCREEN-8**	**66**
**NIS**	**67**
**NRI**	**28**
**G8**	**29**
**MNA**	**68**
**NST**	**65**
**Brief Resilience Scale**	**69**

Supplementary Table S3. Comparison of all data in subgroup divided by hospital intake ≤75% vs. >75% and ≤50% vs. >50%.

		50%≤	50%>	*p* Value	75%≤	75%>	*p*-Value
ICD-10 Version:2016,N(%)		308 (84)	57 (16)		226 (62)	139 (38)	
Categories	Title						
I	Certain infectious and parasitic diseases	2 (1)	0 (0)	0.712	1 (0)	1 (1)	0.617
II	Neoplasms	31 (10)	11 (19)	0.045	21 (9)	21 (15)	0.091
III	Diseases of the blood and blood-forming organs and certain disorders involving the immune mechanism	0 (0)	1 (2)	0.156	0 (0)	1 (1)	0.381
IV	Endocrine, nutritional and metabolic diseases	43 (14)	0 (0)	0.003	39 (17)	4 (3)	< 0.001
VI	Diseases of the nervous system	17 (6)	12 (21)	<0.001	12 (5)	17 (12)	0.018
VII	Diseases of the eye and adnexa	29 (9)	1 (2)	0.035	28 (12)	2 (1)	< 0.001
IX	Diseases of the circulatory system	18 (6)	3 (5)	0.579	10 (4)	11 (8)	0.165
X	Diseases of the respiratory system	5 (2)	0 (0)	0.426	2 (1)	3 (2)	0.284
XI	Diseases of the digestive system	13 (4)	8 (14)	0.009	3 (1)	18 (13)	< 0.001
XII	Diseases of the skin and subcutaneous tissue	2 (1)	0 (0)	0.712	1 (0)	1 (1)	0.617
XIII	Diseases of the musculoskeletal system and connective tissue	54 (18)	0 (0)	< 0.001	44 (20)	10 (7)	0.001
XIV	Diseases of the genitourinary system	33 (11)	3 (5)	0.205	30 (13)	6 (4)	0.005
XVIII	Symptoms, signs and abnormal clinical and laboratory findings, not elsewhere classified	3 (1)	0 (0)	0.600	2 (1)	1 (1)	0.676
XIX	Injury, poisoning and certain other consequences of external causes	10 (3)	1 (2)	0.466	6 (3)	5 (4)	0.414
XXI	Factors influencing health status and contact with health services	48 (16)	17 (30)	0.010	27 (12)	38 (27)	< 0.001

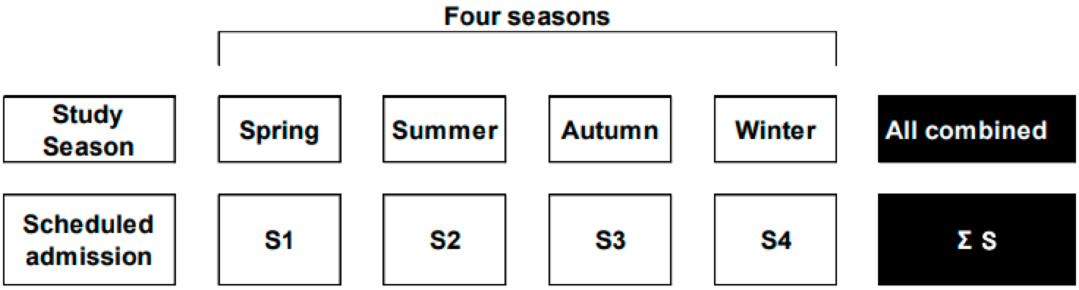

Supplementary Figure S1. Subject distribution. Study Season Spring Summer Autumn Winter All combined Σ S Four seasons Scheduled admission S1 S2 S3 S4 All subjects were divided in to four groups, S1 – S4, by the season of data collection. Abbreviations, S1: S in spring, S2: S in summer, S3: S in autumn, S4: S in winter, Σ S: all S patients combined.

References

26. Martinuzzi ALN, Manzanares W, Quesada E, Reberendo MJ, Baccaro F, Aversa I, et al. Nutritional risk and clinical outcomes in critically ill adult patients with COVID-19. Nutr Hosp. 2021;38(6):1119-1125. doi: 10.20960/nh.03749.
27. Li G, Zhou CL, Ba YM, Wang YM, Song B, Cheng XB, et al. Nutritional risk and therapy for severe and critical COVID-19 patients: A multicenter retrospective observational study. Clin Nutr. 2021 Apr;40(4):2154-2161. doi: 10.1016/j.clnu.2020.09.040.
28. Liu G, Zhang S, Mao Z, Wang W, Hu H. Clinical significance of nutritional risk screening for older adult patients with COVID-19. Eur J Clin Nutr. 2020;74(6):876-883. doi: 10.1038/s41430-020-0659-7.
29. Can B, Senturk Durmus N, Olgun Yıldızeli S, Kocakaya D, Ilhan B, Tufan A. Nutrition risk assessed by Nutritional Risk Screening 2002 is associated with in-hospital mortality in older patients with COVID-19. Nutr Clin Pract. 2022;37(3):605-614. doi: 10.1002/ncp.10860.

30. Zhang K, Gui H, Cong J, He P. A modified nutrition risk screening 2002 predicts the risk of death among hospitalized patients with COVID-19. Clin Nutr ESPEN. 2022;52:365-370. doi: 10.1016/j.clnesp.2022.09.018.
31. Ahmadi S, Firoozi D, Dehghani M, Zare M, Mehrabi Z, Ghaseminasab-Parizi M, et al. Evaluation of Nutritional Status of Intensive Care Unit COVID-19 Patients Based on the Nutritional Risk Screening 2002 Score. Clin Pract. 2022;2022:2448161. doi: 10.1155/2022/2448161.
32. Zhao X, Li Y, Ge Y, Shi Y, Lv P, Zhang J, et al. Evaluation of Nutrition Risk and Its Association With Mortality Risk in Severely and Critically Ill COVID-19 Patients. JPEN J Parenter Enteral Nutr. 2021;45(1):32-42. doi: 10.1002/jpen.1953.
33. Alikiaii B, Heidari Z, Fazeli A, Rahimi Varposhti M, Moradi Farsani D, Fattahpour S, et al. Evaluation of the effectiveness of the Nutritional Risk Screening System 2002 (NRS-2002) in COVID-19 patients admitted to the intensive care unit. Int J Clin Pract. 2021;75(12):e14934. doi: 10.1111/ijcp.14934.
34. Fatemeh G, Fotsing G, Marques-Vidal P, Kopp P, Barigou M. Predictive value of multiple variable models including nutritional risk score (NRS 2002) on mortality and length of stay of patients with covid-19 infections. The INCOVO study. Clin Nutr ESPEN. 202355:357-363. doi: 10.1016/j.clnesp.2023.04.001.
35. Pironi L, Sasdelli AS, Ravaioli F, Baracco B, Battaiola C, Bocedi G, et al. Malnutrition and nutritional therapy in patients with SARS-CoV-2 disease. Clin Nutr. 2021;40(3):1330-1337. doi: 10.1016/j.clnu.2020.08.021.
36. Gregoriano C, Voelkle M, Koch D, Hauser SI, Kutz A, Mueller B, et al. Association of Different Malnutrition Parameters and Clinical Outcomes among COVID-19 Patients: An Observational Study. Nutrients. 2022;14(16):3449. doi: 10.3390/nu14163449.
37. Shamlan G, Albreiki M, Almasoudi HO, Alshehri LA, Ghaith MM, Alharthi AS, et al. Nutritional status of elderly patients previously ill with COVID-19: Assessment with nutritional risk screening 2002 (NRS-2002) and mini nutritional assessment (MNA-sf). J Infect Public Health. 2024;17(2):372-377. doi: 10.1016/j.jiph.2023.11.005. 2002 (NRS-2002) and mini nutritional assessment (MNA-sf).
38. Mendes A, Serratrice C, Herrmann FR, Gold G, Graf CE, Zekry D, et al. Nutritional risk at hospital admission is associated with prolonged length of hospital stay in old patients with COVID-19. Clin Nutr. 2022;41(12):3085-3088. doi: 10.1016/j.clnu.2021.03.017.
39. Kasapoglu US, Gok A, Delen LA, Ozer AB. Comparison of nutritional risk status assessment tools in predicting 30-day survival in critically ill COVID-19 pneumonia patients. Ann Saudi Med. 2022;42(4):236-245. doi: 10.5144/0256-4947.2022.236.
40. Polat O, Yuruyen M, Sonmezoz GB, Kansu AD, Erismis B, Karendere F, et al. Malnutrition risk frequency and independent risk factors associated with mortality in hospitalized elderly patients with COVID-19 in Turkey. Asia Pac J Clin Nutr. 2022;31(3):355-361. doi: 10.6133/apjcn.202209_31(3).0003.
41. Sun Y, He J, Li W, Li S, Lin Y, Cen Y, Li Y. Preliminary Exploration of Setting the Disease Severity Score in the Nutritional Risk Screening of Patients with Severe Novel Coronavirus Pneumonia by Applying NRS2002. Int J Gen Med. 2021;14:1167-1172. doi: 10.2147/IJGM.S289655. eCollection 2021.
42. Eslamian G, Sali S, Babaei M, Parastouei K, Moghadam DA. Association of nutrition risk screening 2002 and Malnutrition Universal Screening Tool with COVID-19 severity in hospitalized patients in Iran. Acute Crit Care. 2022;37(3):332-338. doi: 10.4266/acc.2021.01830.
43. Cuerda C, López IS, Martínez CG, Viveros MM, Impact of COVID-19 in nutritional and functional status of survivors admitted in intensive care units during the first outbreak. Preliminary results of the NUTRICOVID study. Clin Nutr. 2022;41(12):2934-2939. doi: 10.1016/j.clnu.2021.11.017.
44. McGovern J, Al-Azzawi Y, Kemp O, Moffitt P, Richards C, Dolan RD,et al. The relationship between frailty, nutritional status, co-morbidity, CT-body composition and systemic inflammation in patients with COVID-19. J Transl Med. 2022;20(1):98. doi: 10.1186/s12967-022-03300-2.

45. Damanti S, Cilla M, Vitali G, Tiraferri V, Pomaranzi C, De Rubertis G, et al. Exploring the Association between Delirium and Malnutrition in COVID-19 Survivors: A Geriatric Perspective. Nutrients. 202315(22):4727. doi: 10.3390/nu15224727.
46. Riesgo H, Castro A, Del Amo S, San Ceferino MJ, Izaola O, Primo D, et al. Prevalence of Risk of Malnutrition and Risk of Sarcopenia in a Reference Hospital for COVID-19: Relationship with Mortality. Ann Nutr Metab. 2021;77(6):324-329. doi: 10.1159/000519485.
47. Vong T, Yanek LR, Wang L, Yu H, Fan C, Zhou E, et al. Malnutrition Increases Hospital Length of Stay and Mortality among Adult Inpatients with COVID-19. Nutrients. 2022;14(6):1310. doi: 10.3390/nu14061310.
48. Cvijetić S, Keser I, Boschiero D, Ilich JZ. Osteosarcopenic Adiposity and Nutritional Status in Older Nursing Home Residents during the COVID-19 Pandemic. Nutrients. 2023;15(1):227. doi: 10.3390/nu15010227.
49. Kananen L, Eriksdotter M, Boström AM, Kivipelto M, Annetorp M, Metzner C, et al. Body mass index and Mini Nutritional Assessment-Short Form as predictors of in-geriatric hospital mortality in older adults with COVID-19. Clin Nutr. 2022;41(12):2973-2979. doi: 10.1016/j.clnu.2021.07.025.
50. Nguyen LT, Ta TV, Bui AT, Vo SN, Nguyen NLT. Nutritional Status, Refeeding Syndrome and Some Associated Factors of Patients at COVID-19 Hospital in Vietnam. Nutrients. 2023;15(7):1760. doi: 10.3390/nu15071760.
51. Bedock D, Couffignal J, Bel Lassen P, Soares L, Mathian A, Fadlallah JP, et al. Evolution of Nutritional Status after Early Nutritional Management in COVID-19 Hospitalized Patients. Nutrients. 2021 Jun;13(7):2276. doi: 10.3390/nu13072276.
52. Mosbah H, Fadlallah J, Amoura Z, Oppert JM, Faucher P. Prevalence and severity of malnutrition in hospitalized COVID-19 patients. Clin Nutr ESPEN. 2020;40:214-219. doi: 10.1016/j.clnesp.2020.09.018.
53. Gobbi M, Brunani A, Arreghini M, Baccalaro G, Dellepiane D, La Vela V, et al. Nutritional status in post SARS-Cov2 rehabilitation patients. Clin Nutr. 2022;41(12):3055-3060. doi: 10.1016/j.clnu.2021.04.013.
54. Demirkol ME, Aktas G, Alisik M, Yis OM, Kaya M, Kocadag D. Is the prognostic nutritional index a predictor of Covid-19 related hospitalizations and mortality? Malawi Med J. 2023;35(1):15-21. doi: 10.4314/mmj.v35i1.4.
55. Al-Shami I, Hourani HMA, Alkhatib B. The use of prognostic nutritional index (PNI) and selected inflammatory indicators for predicting malnutrition in COVID-19 patients: A retrospective study. J Infect Public Health. 2023;16(2):280-285. doi: 10.1016/j.jiph.2022.12.018.
56. Wei W, Wu X, Jin C, Mu T, Gu G, Min M, Mu S, Han Y. Predictive Significance of the Prognostic Nutritional Index (PNI) in Patients with Severe COVID-1. J Immunol Res. 2021;2021:9917302. doi: 10.1155/2021/9917302. eCollection 2021.
57. Song F, Ma H, Wang S, Qin T, Xu Q, Yuan H, et al. Nutritional screening based on objective indices at admission predicts in-hospital mortality in patients with COVID-19. Nutr J. 2021;20(1):46. doi: 10.1186/s12937-021-00702-8.
58. Sanchez-Rodriguez D, Sacco G, Gautier J, Brière O, Annweiler C; GERIA-COVID study group. Effects of malnutrition on mortality in oldest-old inpatients with COVID-19 in the GERIA-COVID cohort. Maturitas. 2022;161:40-43. doi: 10.1016/j.maturitas.2021.11.016.
59. De Meester D, Goossens M, Marco E, Claessens M, Gautier J, Annweiler C, et al. Evaluation of the Geriatric Nutritional Risk Index in predicting mortality in older patients with COVID-19 in the AgeBru cohort. Clin Nutr ESPEN. 2023t;57:65-72. doi: 10.1016/j.clnesp.2023.06.025.
60. Braga-da-Silveira J, Baron MV, Carati-da-Rocha GG, Reinheimer IC, Figueiredo AE, Poli-de-Figueiredo CE. Nutritional risk and morbidity and mortality in intensive care unit patients with Coronavirus disease 2019. Clin Nutr ESPEN. 2024;60:234-239. doi: 10.1016/j.clnesp.2024.02.002.
61. Kucuk B, Baltaci Ozen S, Kocabeyoglu GM, Mutlu NM, Cakir E, Ozkocak Turan I. NUTRIC Score Is Not Superior to mNUTRIC Score in Prediction of Mortality of COVID-19 Patients. Int J Clin Pract. 2022;2022:1864776. doi: 10.1155/2022/1864776. eCollection 2022.

62. Sendrós MJ, Soldevila B, Puig-Domingo M. Impact of COVID-19 on nutritional status during the first wave of the pandemic. Clin Nutr. 2022;41(12):3032-3037. doi: 10.1016/j.clnu.2021.05.001.
63. Ramos A, Joaquin C, Ros M, Martin M, Cachero M, Sospedra M,et al. Early nutritional risk detection and intervention in COVID-19 hospitalized patients through the implementation of electronic automatized alarms. Endocrinol Diabetes Nutr (Engl Ed). 2024;71(2):71-76. doi: 10.1016/j.endien.2024.03.008.
64. Ali AM, Kunugi H. Approaches to Nutritional Screening in Patients with Coronavirus Disease 2019 (COVID-19). Int J Environ Res Public Health. 2021;18(5):2772. doi: 10.3390/ijerph18052772.
65. Bell J, Heyer N, Greenstein A, Fragkos K, Baxter-Derrington C, Mehta S. A retrospective analysis of malnutrition risk, nutritional support and outcomes in COVID-19 patients. Clin Nutr ESPEN. 2022;50:196-206. doi: 10.1016/j.clnesp.2022.05.017.
66. Wei C, Beauchamp MK, Vrkljan B, Vesnaver E, Giangregorio L, Macedo LG, Keller HH. Loneliness and resilience are associated with nutrition risk after the first wave of COVID-19 in community-dwelling older Canadians. Appl Physiol Nutr Metab. 2023;48(1):38-48. doi: 10.1139/apnm-2022-0201.
67. Fiorindi C, Campani F, Rasero L, Campani C, Livi L, Giovannoni L, Aet al. Prevalence of nutritional risk and malnutrition during and after hospitalization for COVID-19 infection: Preliminary results of a single-centre experience. Clin Nutr ESPEN. 2021;45:351-355. doi: 10.1016/j.clnesp.2021.07.020.
68. Haraj NE, El Aziz S, Chadli A, Dafir A, Mjabber A, Aissaoui O,et al. Nutritional status assessment in patients with Covid-19 after discharge from the intensive care unit. Clin Nutr ESPEN. 2021;41:423-428. doi: 10.1016/j.clnesp.2020.09.214.
69. Capicio M, Panesar S, Keller H, Gramlich L, Popeski N, Basualdo-Hammond C, et al. Nutrition Risk, Resilience and Effects of a Brief Education Intervention among Community-Dwelling Older Adults during the COVID-19 Pandemic in Alberta, Canada. Nutrients. 2022;14(5):1110. doi: 10.3390/nu14051110.
